# Recent Advances in the Management of Non-rheumatic Atrial Fibrillation: A Comprehensive Review

**DOI:** 10.7759/cureus.65835

**Published:** 2024-07-31

**Authors:** Abhinav Kadam, Palash S Kotak, Kashish Khurana, Saket S Toshniwal, Varun Daiya, Sarang S Raut, Sunil Kumar, Sourya Acharya

**Affiliations:** 1 Internal Medicine, Jawaharlal Nehru Medical College, Datta Meghe Institute of Higher Education and Research, Wardha, IND; 2 Medicine, Jawaharlal Nehru Medical College, Datta Meghe Institute of Higher Education and Research, Wardha, IND

**Keywords:** emerging therapies, catheter ablation, pharmacotherapy, management, non-rheumatic, atrial fibrillation

## Abstract

Atrial fibrillation (AF) is a prevalent cardiac arrhythmia characterized by irregular atrial electrical activity, posing significant challenges to patient management and healthcare systems worldwide. Non-rheumatic AF, distinct from AF due to rheumatic heart disease, encompasses a spectrum of etiologies, including hypertension, coronary artery disease, and structural heart abnormalities. This review examines the latest advancements in managing non-rheumatic AF, encompassing diagnostic approaches, pharmacological therapies, and innovative non-pharmacological interventions. Diagnostic strategies ranging from traditional electrocardiography to advanced imaging modalities are explored alongside emerging biomarkers and wearable technologies facilitating early detection and management. Pharmacological management options, including novel anticoagulants and rhythm control agents, are evaluated in light of current guidelines and recent clinical trials. Non-pharmacological interventions, such as catheter ablation and device-based therapies, are discussed regarding their evolving techniques and outcomes. Special considerations for diverse patient populations, including elderly individuals and athletes, are addressed, emphasizing personalized approaches to optimize therapeutic outcomes. The review concludes with insights into future directions for AF management, highlighting promising avenues in gene therapy, regenerative medicine, and precision medicine approaches. By synthesizing recent research findings and clinical innovations, this review provides a comprehensive overview of the dynamic landscape of non-rheumatic AF management, offering insights for clinicians, researchers, and healthcare stakeholders.

## Introduction and background

Atrial fibrillation (AF) is the most prevalent sustained cardiac arrhythmia encountered in clinical practice. Characterized by irregular and often rapid electrical impulses within the atria, AF disrupts the normal rhythm of the heart, compromising its efficiency in pumping blood [1]. Non-rheumatic AF specifically excludes cases where AF results from rheumatic heart disease, focusing instead on underlying causes such as hypertension, coronary artery disease, and structural abnormalities of the heart [2].

The clinical impact of AF extends beyond its prevalence, significantly affecting patient health due to its strong association with increased risks of stroke, heart failure, and overall mortality [3]. The irregular contraction of the atria in AF can lead to blood stasis, predisposing patients to thromboembolic events, particularly ischemic strokes. Furthermore, the hemodynamic instability associated with AF can exacerbate symptoms of heart failure, impairing quality of life and increasing healthcare utilization [4].

This review aims to delve into recent advancements in managing non-rheumatic AF, emphasizing both pharmacological and non-pharmacological interventions. It seeks to offer a comprehensive overview of the evolving landscape in AF management by examining current diagnostic strategies, treatment modalities, and emerging therapies. Additionally, the review will underscore special considerations tailored to diverse patient populations and explore future directions in AF research and therapeutic strategies. By addressing these aspects, the review aims to contribute to optimizing the management of AF, enhancing patient outcomes, and refining clinical approaches to this prevalent and impactful cardiac arrhythmia.

## Review

Pathophysiology of non-rheumatic atrial fibrillation

Mechanisms Underlying Atrial Fibrillation

The onset of non-rheumatic AF typically involves ectopic activity, often originating from the pulmonary veins. These triggers may result from abnormal automaticity or early/delayed afterdepolarizations. However, the mere presence of triggers alone does not sustain AF. Developing an arrhythmogenic substrate is also essential for perpetuating this intricate arrhythmia [5]. The AF substrate encompasses both electrical and structural remodeling of the atria. Electrical remodeling, characterized by alterations in ion channels and action potentials, creates an environment prone to initiating and maintaining AF. Shortening the atrial effective refractory period and action potential duration facilitates the establishment of reentry circuits [6]. Structural remodeling, particularly atrial fibrosis, introduces heterogeneity and slowed conduction, further promoting reentry. Fibrosis establishes a foundation for multiple wavelets and reentry circuits. Imbalance in the autonomic nervous system can also contribute to the AF substrate by modifying atrial electrophysiological properties [7]. Once initiated, AF can persist through either reentrant mechanisms or rapid, sustained ectopic activity. Reentry relies on a trigger, unidirectional block, and a critical tissue mass. Electrical and structural remodeling foster the endurance of AF over time, driving the progression from paroxysmal to persistent and permanent forms of arrhythmia. Understanding these intricate mechanisms underlying AF initiation and perpetuation is critical for developing more effective therapies to prevent and manage this prevalent and often debilitating condition [8].

Risk Factors and Associated Conditions

**Figure 1:**
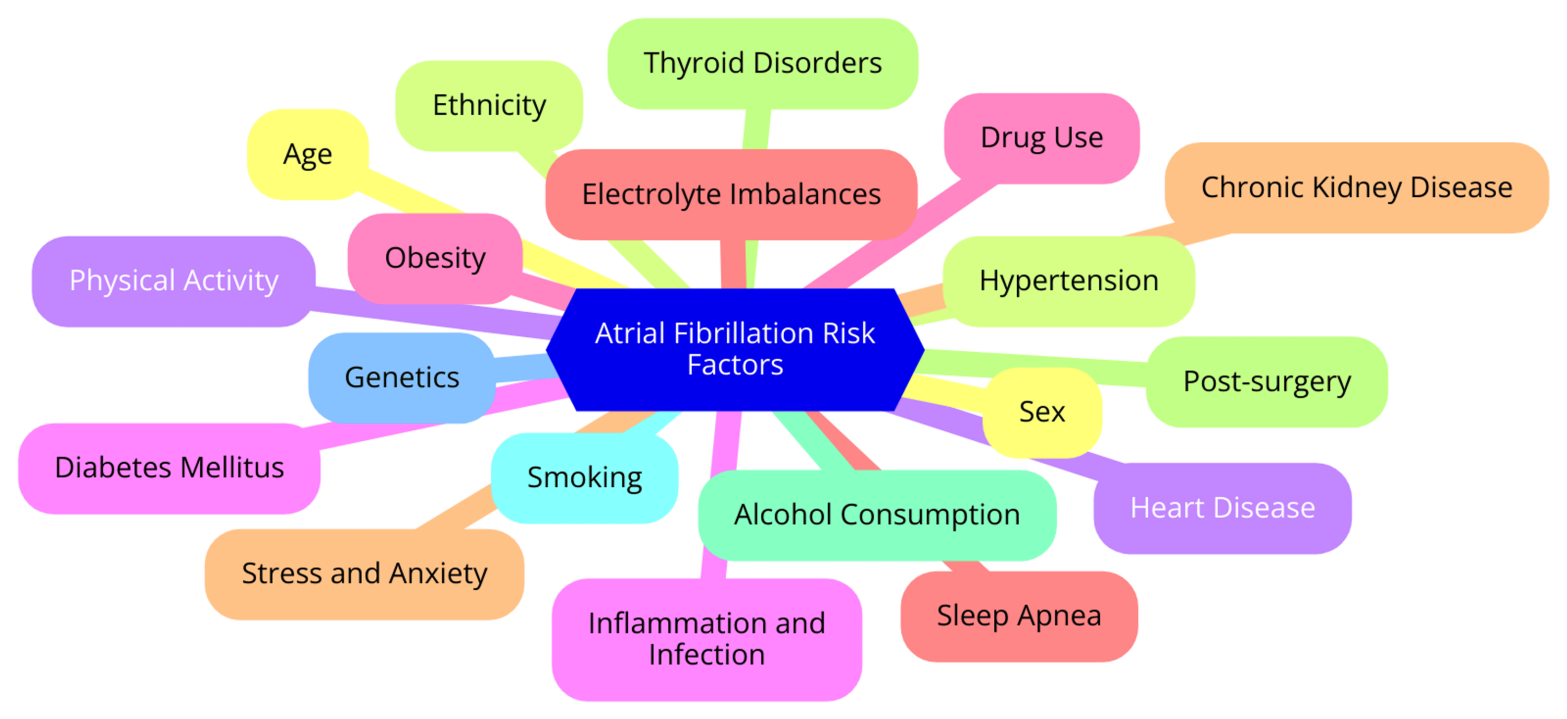
Risk factors and associated conditions of atrial fibrillation. Image credit: Dr. Abhinav Kadam.

Non-rheumatic AF is closely associated with a range of cardiovascular risk factors. Hypertension, coronary artery disease, and heart failure are among the primary contributors capable of inducing both structural and electrical changes in the atria. Lifestyle behaviors such as excessive alcohol consumption, smoking, and obesity also significantly contribute to the development of this arrhythmia. Additionally, conditions such as sleep apnea, thyroid disorders, and pulmonary diseases have been linked to an elevated risk of non-rheumatic AF [9]. Beyond its risk factors, non-rheumatic AF is also closely linked to serious health implications. Patients with this condition face an increased risk of ischemic stroke, exhibiting higher mortality rates and more severe brain damage compared to those without AF. The irregular and rapid heart rate associated with non-rheumatic AF can progressively weaken the heart muscle, leading to an elevated risk of heart failure and tachycardia-mediated cardiomyopathy. Moreover, the arrhythmia significantly heightens the risk of thromboembolism, particularly originating from the left atrial appendage [10]. Understanding the intricate interplay between these risk factors and associated conditions is crucial for effectively managing non-rheumatic AF. By addressing the underlying causes driving this arrhythmia and managing its potential complications, healthcare providers can enhance overall outcomes and improve the quality of life for patients grappling with this challenging condition [11]. Risk factors and associated conditions of AF are shown in Figure 1.

Genetic and Molecular Basis

Non-rheumatic AF exhibits a significant genetic component, with approximately 30% of patients having a familial history of the condition. Genome-wide association studies have identified over 140 genetic loci linked to AF risk. These common variants typically affect ion channels, transcription factors, cytoskeletal proteins, and other pathways crucial for atrial electrical and structural remodeling. Additionally, rare genetic variants in ion channel genes, such as *KCNQ1*, *KCNA5*, *SCN5A*, and genes encoding non-ion channel proteins, such as *NKX2-5*, *GATA4*, and *PITX2*, have been associated with familial and early-onset forms of AF [12]. The genetic variants associated with AF disrupt normal atrial electrophysiology and structure through several pathways. These include changes in ion channel function leading to abnormal action potentials and conduction, dysregulation of transcription factors and signaling pathways involved in atrial remodeling, and defects in cytoskeletal and structural proteins resulting in atrial fibrosis and dilation. These molecular alterations create a substrate predisposed to initiating and perpetuating abnormal electrical activity observed in AF [13]. Understanding the genetic and molecular foundations of AF is pivotal for tailoring personalized treatment strategies. This may involve targeted therapies based on specific genetic variants, selecting antiarrhythmic drugs (AADs) or ablation techniques guided by genotype, and employing polygenic risk scores to refine risk assessment and treatment decisions. Ongoing research in this field promises to advance more personalized and effective management approaches for this prevalent arrhythmia [14].

Diagnostic approaches

Clinical Evaluation and History-Taking

Clinical evaluation of non-rheumatic AF commences with a comprehensive history assessment. It is crucial to ascertain whether the AF episodes are paroxysmal or persistent and to detail associated symptoms. Patients should be queried about potential triggers, underlying heart conditions contributing to the arrhythmia, and any precipitating factors. Clinicians must also investigate symptoms indicative of heart failure, such as dyspnea, orthopnea, and edema, which are common in AF patients. Inquiring about risk factors such as hypertension, valvular heart disease, and cardiomyopathy is essential, as is evaluating for AF-related complications such as stroke, thromboembolism, and mortality [15]. The physical examination forms another vital aspect of the clinical evaluation. Blood pressure and heart rate measurements are standard, and auscultation for cardiac murmurs suggestive of valvular abnormalities is important. Signs indicating heart failure, such as pulmonary rales, S3 gallop, peripheral pulses, and jugular venous distention, should also be carefully assessed [16]. Risk stratification constitutes a pivotal step in evaluating patients with non-rheumatic AF. The CHA_2_DS_2_-VASc score aids in assessing stroke risk, while the Outcomes Registry for Better Informed Treatment (ORBIT) score is valuable for evaluating bleeding risk when contemplating anticoagulation therapy. Identification and management of modifiable bleeding risk factors, such as uncontrolled hypertension, labile international normalized ratio, and concurrent medications, are critical components of this process [17].

Role of Electrocardiography and Holter Monitoring

Electrocardiography (ECG) is the primary diagnostic tool for detecting and confirming the presence of AF. It effectively identifies the characteristic irregular and chaotic electrical activity patterns associated with AF, including the absence of P-waves and irregular R-R intervals. ECG provides direct evidence essential for the initial diagnosis of AF [18]. In cases where the initial ECG does not reveal AF despite strong clinical suspicion, Holter monitoring may be warranted. Holter monitoring enables continuous cardiac rhythm assessment over an extended period, facilitating the detection of paroxysmal or intermittent AF episodes that may not be captured during a single ECG recording. This monitoring method yields valuable insights into the frequency, duration, and patterns of AF episodes, which are crucial for guiding treatment decisions [19]. ECG and Holter monitoring play complementary roles in comprehensively evaluating non-rheumatic AF. While ECG confirms the presence of AF and its initial diagnosis, Holter monitoring is instrumental in identifying intermittent episodes that might be missed initially. These diagnostic modalities are indispensable for accurately diagnosing and characterizing the type and patterns of AF, thereby informing appropriate and tailored treatment strategies [20].

Advanced Imaging Techniques

Advanced imaging techniques are critical in diagnosing and managing cardiac conditions, offering detailed insights into cardiac structure and function. Echocardiography, a fundamental tool, encompasses traditional methods such as transthoracic echocardiography, which uses an external probe, and transesophageal echocardiography, providing more detailed images via an esophageal probe [21]. Advanced echocardiographic techniques further enhance assessment capabilities. Three-dimensional echocardiography offers comprehensive views of cardiac structures from all angles, improving accuracy in evaluation. Speckle tracking echocardiography monitors heart muscle movement to assess cardiac strain and function. Contrast echocardiography uses contrast agents to highlight subtle cardiac abnormalities [22]. Cardiac magnetic resonance imaging (MRI) utilizes magnetic fields and radio waves to produce high-resolution images, which are ideal for assessing cardiac structure and function and detecting conditions such as cardiac tumors and coronary artery disease. Stress testing with MRI can evaluate cardiac performance under varied conditions, aiding in coronary artery disease diagnosis [23]. Cardiac computed tomography (CT) employs X-rays and computer technology to generate detailed heart and blood vessel images, crucial for diagnosing coronary artery disease and evaluating cardiac structures. Calcium scoring via cardiac CT assesses coronary artery calcium levels, helping gauge coronary disease risk [24]. Nuclear cardiology techniques such as single-photon emission SPECT and positron emission tomography use radioactive tracers to create images, facilitating coronary artery disease diagnosis and cardiac function assessment [25]. These advanced imaging modalities collectively contribute to comprehensive cardiac evaluation, guiding effective treatment strategies and enhancing patient care.

Emerging Diagnostic Tools and Biomarkers

Wearable devices such as smartwatches and fitness trackers are revolutionizing the detection and monitoring of AF. These devices utilize photoplethysmography and other sensors to continuously monitor heart rhythm, identify irregular heartbeats, and offer a convenient AF screening and tracking method. Studies have shown that wearable devices can achieve high accuracy in detecting AF compared to standard ECG monitoring, marking them as promising tools for early detection and management of this arrhythmia [26]. Artificial intelligence (AI) and machine learning algorithms are also pivotal in advancing AF diagnosis. These automated tools can analyze data from wearable devices or ECGs, detecting patterns indicative of AF with impressive sensitivity and specificity. By leveraging AI, clinicians can streamline the diagnostic process and enhance early AF detection, potentially improving patient outcomes [27]. In addition to technological innovations, certain blood-based biomarkers are emerging as valuable tools in managing non-rheumatic AF. Elevated levels of natriuretic peptides and cardiac troponins can provide critical insights into underlying structural heart disease or myocardial injury, which are common in AF patients. Integrating biomarker testing into diagnostic protocols helps risk-stratify patients and guides treatment decisions, optimizing care for individuals with this complex arrhythmia [28]. Looking forward, the integration of wearable devices, AI-driven algorithms, and novel cardiac biomarkers promises to transform approaches to diagnosing and managing non-rheumatic AF. By harnessing these emerging tools, clinicians can enhance their ability to detect, monitor, and stratify risk for AF patients, ultimately improving outcomes and quality of life for those affected by this prevalent and challenging condition [29].

Pharmacological management

Anticoagulation Therapy: Current Guidelines and New Anticoagulants

Anticoagulation plays a critical role in stroke prevention for patients with AF, irrespective of the chosen rhythm or rate control strategy. Current guidelines recommend initiating anticoagulation with aspirin, warfarin, or newer oral anticoagulants (NOACs) such as apixaban, dabigatran, edoxaban, and rivaroxaban for all AF patients, except those with contraindications. The selection of anticoagulants should consider the patient’s stroke risk, which is assessed using the CHA_2_DS_2_-VASc score and balanced against the bleeding risk. NOACs are generally preferred over warfarin due to their improved safety and efficacy profiles [30]. Newer NOACs have proven to be effective alternatives to warfarin in stroke prevention for AF patients. Extensive clinical trials have demonstrated that agents such as apixaban, dabigatran, edoxaban, and rivaroxaban are either non-inferior or superior to warfarin, offering enhanced safety profiles and reduced risks of intracranial hemorrhage. These NOACs provide the advantages of fixed dosing, fewer interactions with other drugs, and do not require routine coagulation monitoring. However, they also come with limitations, such as the absence of specific reversal agents for all except dabigatran and higher costs compared to warfarin [31]. For patients with device-detected atrial high-rate episodes but without a prior AF diagnosis, consideration of anticoagulation is based on their stroke risk score. Current guidelines emphasize the pivotal role of anticoagulation in preventing strokes in AF, with NOACs being preferred over warfarin in most patients due to their superior safety and efficacy profiles [32].

Rate Control vs. Rhythm Control Strategies

Choosing between a rhythm and rate control strategy is crucial in managing non-rheumatic AF. Recent evidence indicates that, in most patients, a rhythm control approach using AADs or catheter ablation does not necessarily improve survival or reduce stroke risk compared to a rate control strategy. However, restoring and maintaining sinus rhythm can benefit certain individuals, including improved cardiac hemodynamics, exercise tolerance, and quality of life [11]. The decision to pursue rhythm or rate control should be personalized based on various patient-specific factors. These factors include the patient’s symptom severity, the likelihood of successful cardioversion, and underlying health conditions. For instance, younger patients with symptomatic, recently diagnosed AF and minimal structural heart disease may benefit more from a rhythm control strategy. Conversely, older patients with longstanding AF and multiple comorbidities may find a rate control approach more suitable [33]. Interestingly, the timing of when rhythm control is initiated impacts outcomes significantly. Studies have demonstrated that initiating rhythm control treatment within the first year of an AF diagnosis was associated with reduced risks of cardiovascular death, stroke, heart failure, hospitalization, and myocardial infarction compared to a rate control strategy. However, delaying rhythm control initiation beyond the first year did not provide the same benefits. This underscores the potential advantage of early intervention with a rhythm control approach, particularly for specific patient populations [34].

Antiarrhythmic Drugs: Efficacy, Safety, and Recent Developments

AADs play a central role in managing cardiac arrhythmias, although their efficacy is moderate compared to newer treatments such as catheter ablation. The choice between rhythm control using AADs versus rate control should be personalized based on factors such as symptom severity, likelihood of successful cardioversion, and comorbidities. Recent studies indicate that, for most patients, a rhythm control strategy with AADs or ablation does not necessarily improve survival or reduce stroke risk compared to a rate control approach. However, maintaining sinus rhythm with AADs can enhance cardiac hemodynamics, exercise tolerance, and quality of life, particularly in individuals with new-onset AF and fewer comorbidities [35]. AADs are associated with proarrhythmic effects and other serious adverse events, presenting significant challenges in their clinical use. Close monitoring is essential due to potential interactions with other medications and side effects such as bradycardia, hypotension, and QT prolongation. Recent trials have shown a comparable incidence of adverse effects between AADs and catheter ablation, with AADs demonstrating a trend toward a lower rate of adverse events [36]. The development of new AADs has declined in recent years, partly due to the success of ablation therapy and increased regulatory scrutiny. However, promising advancements include repurposing existing drugs for new therapeutic uses and exploring novel formulations and delivery methods. Researchers are also investigating atrial-selective AADs and combination therapies to enhance effectiveness and safety. Despite ongoing challenges, these developments offer renewed optimism for improving pharmacological treatment options for patients with cardiac arrhythmias [35].

Personalized Medicine and Pharmacogenomics in Atrial Fibrillation Management

Personalized medicine and pharmacogenomics are transforming the management of AF, aiming to tailor therapies based on individual patient characteristics to enhance outcomes and optimize treatment strategies. These approaches integrate genetic profiling, biomarkers, advanced imaging, and mapping technologies to refine risk assessment and guide therapeutic decisions [37]. Genetic variants linked to AF susceptibility, such as those in genes like *KCNH2* and *PITX2*, are being incorporated into risk models to improve the accuracy of predicting AF development and progression. Biomarkers such as high-sensitivity C-reactive protein and N-terminal pro-B-type natriuretic peptide promise to predict AF outcomes and guide treatment approaches. Advanced imaging techniques such as cardiac MRI and CT provide valuable insights into atrial structure, assessing factors such as left atrial size, fibrosis, and fibrotic burden, which are crucial for risk stratification and identifying candidates for specific interventions or therapies [12]. Catheter ablation, particularly pulmonary vein isolation, effectively restores and maintains sinus rhythm in AF patients. Personalized medicine approaches help identify patients likely to benefit most from ablation based on factors such as atrial substrate characteristics, left atrial size, and symptom burden. Pharmacogenetic testing aids in optimizing anticoagulation therapy by identifying patients prone to adverse drug reactions or altered drug metabolism, guiding the choice between vitamin K antagonists and direct oral anticoagulants based on age, renal function, and comorbidities [38]. Anticoagulation therapy remains essential for stroke prevention in AF, balancing stroke and bleeding risks as assessed by the CHA_2_DS_2_-VASc score. The selection of AADs is personalized, considering patient symptoms, underlying heart conditions, drug efficacy, adverse effects, contraindications, pharmacologic interactions, and cost. Factors such as the type of AF (paroxysmal, persistent, or permanent) also influence the choice of antiarrhythmic medication [39].

Non-pharmacological interventions

Catheter Ablation: Techniques, Outcomes, and Innovations

Catheter ablation is a minimally invasive procedure utilized to treat various heart arrhythmias, prominently including AF. Techniques employed in AF catheter ablation encompass pulmonary vein isolation (PVI), radiofrequency ablation (RFA), and cryoablation. PVI aims to electrically isolate pulmonary veins to prevent abnormal impulses triggering AF, guided by three-dimensional (3D) mapping systems ensuring precise catheter placement and vein isolation. RFA involves delivering radiofrequency energy point-by-point to create lesions around pulmonary veins and atrial tissue, which is beneficial for treating persistent or recurrent AF post-PVI. Cryoablation, in contrast, uses extreme cold to freeze and eliminate arrhythmia-causing cells, often combined with PVI to enhance efficacy [40]. Advancements in catheter technology, such as contact force catheters, have enhanced ablation lesion quality and consistency by providing feedback on applied force to the atrial wall. Emerging technologies such as 3D electroanatomic mapping and intracardiac echocardiography further improve procedural precision and safety [41]. Catheter ablation for AF demonstrates promising outcomes, with high success rates in restoring normal heart function, particularly in paroxysmal or persistent AF cases. While AF recurrence post-PVI is possible, the procedure remains valuable, with additional ablation lines augmenting outcomes. Catheter ablation significantly improves symptoms and quality of life, particularly in patients unresponsive to medications [42]. Continued innovations in AF catheter ablation explore novel energy sources such as laser and ultrasound alongside advancements in robotic-assisted catheter navigation and substrate-based ablation targeting specific heart areas. Integrating advanced mapping systems with MRI and CT scans further enhances procedural precision and effectiveness [43]. These ongoing advancements underscore the evolving landscape of AF treatment, aiming to improve patient outcomes and expand therapeutic options.

Surgical Approaches: Maze Procedure and Hybrid Techniques

The maze procedure and hybrid techniques represent significant surgical approaches for managing AF. The classic maze procedure involves creating precise scar tissue patterns in both atria using a scalpel, effectively blocking abnormal electrical signals that cause AF. This traditional “cut and sew” method can be performed via open-heart surgery using a sternotomy incision or through minimally invasive thoracoscopic techniques. Additionally, during the maze procedure, the left atrial appendage, a common site for blood clot formation, is often addressed by removal or closure to reduce stroke risk [44]. Hybrid maze or mini-maze procedures integrate surgical and catheter-based methods to achieve the maze pattern. For instance, the thoracoscopic endoscopic maze (TTm MAZE) is a two-stage procedure. Initially, a cardiac surgeon creates external heart lesions through small incisions using minimally invasive techniques. Subsequently, an electrophysiologist performs catheter ablation to create additional lesions internally, typically around six weeks later. Another hybrid approach is the convergent procedure, combining surgical and catheter ablation components to strategically induce scar tissue formation and restore normal heart rhythm [45]. The selection among open surgical, minimally invasive, or hybrid approaches depends on factors such as the type and severity of AF, previous treatments, and concurrent heart conditions. These procedures are particularly valuable for AF patients who have not responded to other therapies or who require surgical intervention for associated heart disease [46]. The maze procedure and hybrid techniques thus offer important treatment options to manage AF and improve patient outcomes effectively.

Device Therapy: Pacemakers and Implantable Cardioverter-Defibrillators

Device therapy, particularly the use of pacemakers and implantable cardioverter-defibrillators (ICDs), plays a crucial role in managing cardiac arrhythmias and heart failure. Pacemakers are small, battery-powered devices implanted in the body to regulate heart rate by delivering electrical impulses to the heart muscle. They are utilized primarily for treating bradycardia (slow heart rate) and other cardiac rhythm disorders. Pacemakers can be single-chamber, dual-chamber, or biventricular (cardiac resynchronization therapy, CRT), depending on the patient’s needs [47]. The implantation of a pacemaker involves a surgical procedure where wires (leads) are inserted into a major vein under the collarbone and guided to the heart using X-ray images. These leads connect to the pacemaker, positioned under the skin near the collarbone. Pacemakers can enhance quality of life by stabilizing abnormal heart rhythms, preventing life-threatening conditions, and aiding in managing heart failure by improving cardiac function and reducing symptoms. However, pacemakers necessitate periodic battery replacement surgeries, which can introduce complications and impact the quality of life. Patients with pacemakers must also avoid activities and environments that could interfere with the device’s function, such as MRI scans [48].

ICDs are devices designed to detect and treat life-threatening arrhythmias such as ventricular tachycardia and ventricular fibrillation by delivering electrical shocks to restore a normal heart rhythm. ICDs can be single-chamber or dual-chamber, tailored to meet individual patient needs. Like pacemakers, ICDs are implanted with leads inserted into the heart and connected to the device. They serve to prevent sudden cardiac death by promptly detecting and correcting life-threatening arrhythmias. Often used in conjunction with pacemakers, ICDs provide comprehensive cardiac rhythm management. However, like pacemakers, ICDs require periodic battery replacements and necessitate precautions against certain activities and environments that could interfere with device functionality [49]. CRT is a specialized pacemaker that coordinates electrical signals to both lower heart chambers, synchronizing their contractions to enhance cardiac function and alleviate symptoms in patients with heart failure. CRT devices are implanted similarly to other pacemakers, with leads inserted into the heart and connected to the device. CRT can significantly improve cardiac function and symptomatology, particularly in patients with left bundle branch block. However, CRT also requires periodic battery replacement surgeries, and patients must avoid activities and environments that could disrupt the device’s operation [50].

Lifestyle Modifications and Risk Factor Management

Lifestyle modifications and managing risk factors are pivotal in preventing and managing AF. Even modest reductions in body weight, such as 10%, can alleviate AF symptoms and lower AF risk. Maintaining a healthy body mass index is crucial for AF prevention. Engaging in moderate-intensity aerobic exercise for at least 150 minutes per week is recommended by the American Heart Association to prevent AF and enhance symptoms in those with existing AF [51]. Treating obstructive sleep apnea (OSA) with continuous positive airway pressure devices can significantly improve AF symptoms. Adjusting sleep positions to reduce airway blockages also aids in managing OSA, thus benefiting AF management. Cutting down on alcohol consumption or abstaining altogether can help alleviate AF symptoms and reduce recurrence. Quitting smoking is vital for decreasing AF risk and other cardiovascular ailments.

Adopting a diet low in salt, saturated fats, trans fats, and cholesterol supports AF prevention and enhances cardiovascular health. Engaging in stress-reducing activities such as yoga, meditation, or acupuncture helps manage stress, a recognized trigger for AF [52]. Maintaining controlled blood pressure and managing diabetes is essential for reducing AF risk and improving overall cardiovascular health. Additionally, managing cholesterol levels through dietary adjustments and medications contributes to AF prevention. Understanding and addressing genetic risk factors can also aid in preventing AF and improving cardiovascular health. Avoiding excessive caffeine and stimulants, including over-the-counter medications containing stimulants, helps minimize AF triggers. Regular monitoring and managing underlying conditions such as hypertension, diabetes, and sleep apnea are crucial for AF prevention and cardiovascular health maintenance [53].

Recent technological advances

Wearable Devices and Remote Monitoring

Recent technological advances, particularly smartphones and wearable devices utilizing photoplethysmography, have revolutionized the detection and management of non-rheumatic AF. These tools allow for long-term, non-invasive screening, which enhances the identification of asymptomatic or paroxysmal AF. Continuous monitoring of patient health data through these mobile technologies enables early detection of abnormalities, facilitating prompt interventions. Smartphone-based ECG devices, for instance, offer diagnostic capabilities without requiring additional testing, thereby expanding access to AF diagnosis. However, challenges persist in accessing expert ECG readers in some regions, underscoring the need for further advancements in automated interpretation algorithms [54]. Technological progress in catheter ablation techniques, including advancements in pulmonary vein isolation, has significantly improved rhythm control strategies for symptomatic patients with paroxysmal AF. These innovations have led to more durable procedures and increased success rates over the long term. Combined with the benefits of remote patient monitoring, such minimally invasive interventions support proactive AF management, reducing reliance on frequent hospital visits [55].

Another critical advancement in AF management is the widespread adoption of non-vitamin K antagonist oral anticoagulants (NOACs). These newer agents have substantially lowered the risk of stroke, systemic embolism, and mortality compared to traditional warfarin while maintaining a similar risk profile for major bleeding. Their inclusion in the WHO Essential Medicines List has enhanced global access to effective anticoagulation therapies, particularly in resource-constrained settings. Remote monitoring further optimizes the management of anticoagulant therapy, enabling healthcare providers to monitor patient adherence and response closely [30]. By integrating these technological advancements as mobile devices, wearables, advanced catheter ablation techniques, and the management of non-rheumatic AF is poised for transformation. Continuous monitoring and early detection provided by these innovations can increase patient engagement, improve care coordination, and, ultimately, yield better clinical outcomes for individuals living with this complex cardiac condition [56].

Advances in Catheter Technology and Mapping Systems

Catheter ablation, particularly PVI, has emerged as a highly effective strategy for managing rhythm in symptomatic patients with paroxysmal AF. Recent trials have demonstrated comparable outcomes between pulmonary vein isolation alone and more extensive ablation approaches. Advancements in catheter-based technologies, including enhanced designs, materials, and navigation systems, have been pivotal in achieving durable treatment results [55]. New catheter mapping systems featuring advanced imaging and navigation capabilities, such as 3D imaging, computer-assisted navigation, and real-time catheter tracking, have significantly improved the precision and efficacy of AF treatment. Enhanced mapping catheters with higher electrode density and sophisticated signal processing algorithms provide detailed electrical data of the atria, aiding in identifying and targeting critical AF substrates [57]. Integrating catheter ablation with other technologies such as mobile devices and wearables has enhanced the detection of asymptomatic or paroxysmal AF, enabling earlier intervention and improved management. The availability and adoption of non-vitamin K antagonist oral anticoagulants (NOACs) have also been pivotal advancements, offering significant reductions in stroke risk and mortality compared to traditional warfarin therapy for AF patients [58]. These advancements collectively represent a significant stride forward in the comprehensive management of non-rheumatic AF, improving outcomes and quality of life for affected individuals.

Artificial Intelligence and Machine Learning in AF Management

AI models leveraging deep learning have demonstrated promising capabilities in accurately detecting AF across diverse data sources. These models identify AF from 12-lead ECGs, single-lead ECGs, and even chest radiographs, achieving high accuracy with the area under the curve (AUC) reaching up to 0.81. This suggests that AI holds significant potential to enhance the efficiency and precision of AF diagnosis [27]. In addition to detecting known AF, AI and machine learning techniques applied to electronic health records have improved risk prediction models for future AF development. Studies have reported AUCs as high as 0.827 for predicting future AF using AI models, surpassing traditional clinical risk scores. This advancement could enable earlier intervention and preventive strategies for high-risk individuals [27]. AI models also contribute to stratifying risks among patients with existing AF, predicting outcomes such as stroke, bleeding, and mortality. While the extent of added value over conventional risk scores varies, AI can identify high-risk patients more accurately, potentially guiding more intensive monitoring and treatment approaches [59]. Moreover, AI-powered decision support tools are being explored to assist clinicians in optimizing AF management. For instance, AI may aid in determining anticoagulation dosages or predicting the success of cardioversion procedures. Nonetheless, challenges persist in fully automating complex clinical decision-making using AI, necessitating further research to validate improved patient outcomes in real-world clinical settings [27]. These advancements underscore the evolving role of AI in transforming the diagnosis, prediction, and management of non-rheumatic AF, potentially enhancing patient care and outcomes.

## Conclusions

Non-rheumatic AF management has witnessed significant advancements, offering patients more effective and personalized treatment options. From the refinement of anticoagulation strategies to the evolution of catheter ablation techniques and the integration of innovative technologies, such as wearable devices and AI, the landscape of AF management continues to expand. These developments aim to improve patient outcomes by reducing stroke risk, enhancing quality of life, and mitigating the economic burden associated with AF-related healthcare utilization. However, challenges remain, including the need for further research into optimal treatment sequencing, integrating genetic insights into therapy, and enhancing patient adherence to treatment regimens. As we move forward, continued collaboration between clinicians, researchers, and industry stakeholders will be crucial in advancing the field and achieving better outcomes for individuals living with non-rheumatic AF.
